# Spontaneous improvement of carbohydrate-deficient transferrin in PMM2-CDG without mannose observed in CDG natural history study

**DOI:** 10.1186/s13023-021-01751-2

**Published:** 2021-02-25

**Authors:** Peter Witters, Andrew C. Edmondson, Christina Lam, Christin Johnsen, Marc C. Patterson, Kimiyo M. Raymond, Miao He, Hudson H. Freeze, Eva Morava

**Affiliations:** 1 Department of Paediatrics and Metabolic Center, University Hospitals Leuven, Herestraat 49, 3000 Leuven, Belgium; 2 Department of Development and Regeneration, KU Leuven, Leuven, Belgium; 3 Department of Pediatrics, Division of Human Genetics, Children’s Hospital of Philadelphia, Philadelphia, PA USA; 4 Department of Pediatrics, Division of Genetic Medicine, University of Washington, Seattle, WA USA; 5 Center of Integrated Brain Research, Seattle Children’s Research Institute, Seattle, WA USA; 6 Department of Clinical Genomics, Mayo Clinic, 200 First Street, SW, Rochester, MN 55905 USA; 7 Department of Neurology, Mayo Clinic, Rochester, MN USA; 8 Biochemical Genetics Laboratory, Department of Laboratory Medicine and Pathology, Mayo Clinic, Rochester, MN USA; 9 Department of Pathology and Laboratory Medicine, Children’s Hospital of Philadelphia, Philadelphia, PA USA; 10 Human Genetics Program, Sanford Burnham Prebys Medical Discovery Institute, La Jolla, CA USA

**Keywords:** Phosphomannomutase 2, Congenital disorders of glycosylation, PMM2-CDG, Natural history study, Transferrin, Biomarker

## Abstract

A recent report on long-term dietary mannose supplementation in phosphomannomutase 2 deficiency (PMM2-CDG) claimed improved glycosylation and called for double-blind randomized study of the dietary supplement in PMM2-CDG patients. A lack of efficacy of short-term mannose supplementation in multiple prior reports challenge this study’s conclusions. Additionally, some CDG types have previously been reported to demonstrate spontaneous improvement in glycosylated biomarkers, including transferrin. We have likewise observed improvements in transferrin glycosylation without mannose supplementation. This observation questions the reliability of transferrin as a therapeutic outcome measure in clinical trials for PMM2-CDG. We are concerned that renewed focus on mannose therapy in PMM2-CDG will detract from clinical trials of more promising therapies. Approaches to increase efficiency of clinical trials and ultimately improve patients’ lives requires prospective natural history studies and identification of reliable biomarkers linked to clinical outcomes in CDG. Collaborations with patients and families are essential to identifying meaningful study outcomes.

**Supplementary Information:**

The online version contains supplementary material available at 10.1186/s13023-021-01751-2.

**Dear Editor,**

We read with great interest the article entitled “Dietary mannose supplementation in phosphomannomutase 2 deficiency (PMM2-CDG)” by Taday et al. [1]. PMM2-CDG is a severe multisystemic disease with only supportive treatments currently available [2]. As professionals dedicated to improving the lives of patients with congenital disorders of glycosylation (CDG), we are optimistic about any therapeutic approach that has real promise for altering disease course. Unfortunately, we are concerned that the report by Taday et al.does not provide evidence for a clinical benefit of mannose therapy in PMM2-CDG.

Despite being the most prevalent type of CDG, with an estimated 900 patients worldwide, PMM2-CDG remains ultra-rare. Typical for these diseases, randomized clinical trials are challenging to conduct and clinical decisions are often based on case series reports such as the one by Taday et al. that lack a control group. Carefully compiled natural history studies can provide an important historical control group for comparison of the findings of such studies. Two large retrospective natural history studies including 96 [3] and 75 [4] PMM2-CDG patients have recently been reported, but are limited by their retrospective design. This is a deficiency that our Frontiers in CDG Consortium, a member of the Rare Disease Clinical Research Network, is actively working to rectify through a prospective, multi-institutional natural history study funded by the United States National Institute of Health (https://clinicaltrials.gov/NCT04199000).

We undertook a retrospective analysis of carbohydrate deficient transferrin analysis in PMM2-CDG patients managed at our clinical consortium sites to attempt to provide a natural history context for Taday et al.’s claims that (i) long-term therapy with mannose leads to biochemical improvement including (ia) glycosylation and (ib) coagulation variables and that this (ii) suggests clinical improvement.

## (ia) Improvement of glycosylation

We undertook a retrospective analysis of clinically performed carbohydrate deficient transferrin analysis results of patients followed at Mayo clinic (Rochester, Minnesota, USA), Seattle Children's Hospital (Seattle, Washington, USA), and Children’s Hospital of Philadelphia (Philadelphia, Pennsylvania, USA). These were generally performed by mass spectrometry (MS) analysis. We included all available carbohydrate deficient transferrin analysis results (average 3 measurements/patient, range 1–7 measurements). This resulted in 108 observations in 37 patients (13 females, 24 males, age range 0.6 months—70 years old, see supplemental table for patient and individual measurement details), none of whom were on mannose supplementation. These observations were clustered in younger patients, of which 70 observations were before the age of 100 months, an important note as all of the ‘responders’ in Taday et al.’s report were < 100 months of age when initiating mannose supplementation.

**Fig. 1:**
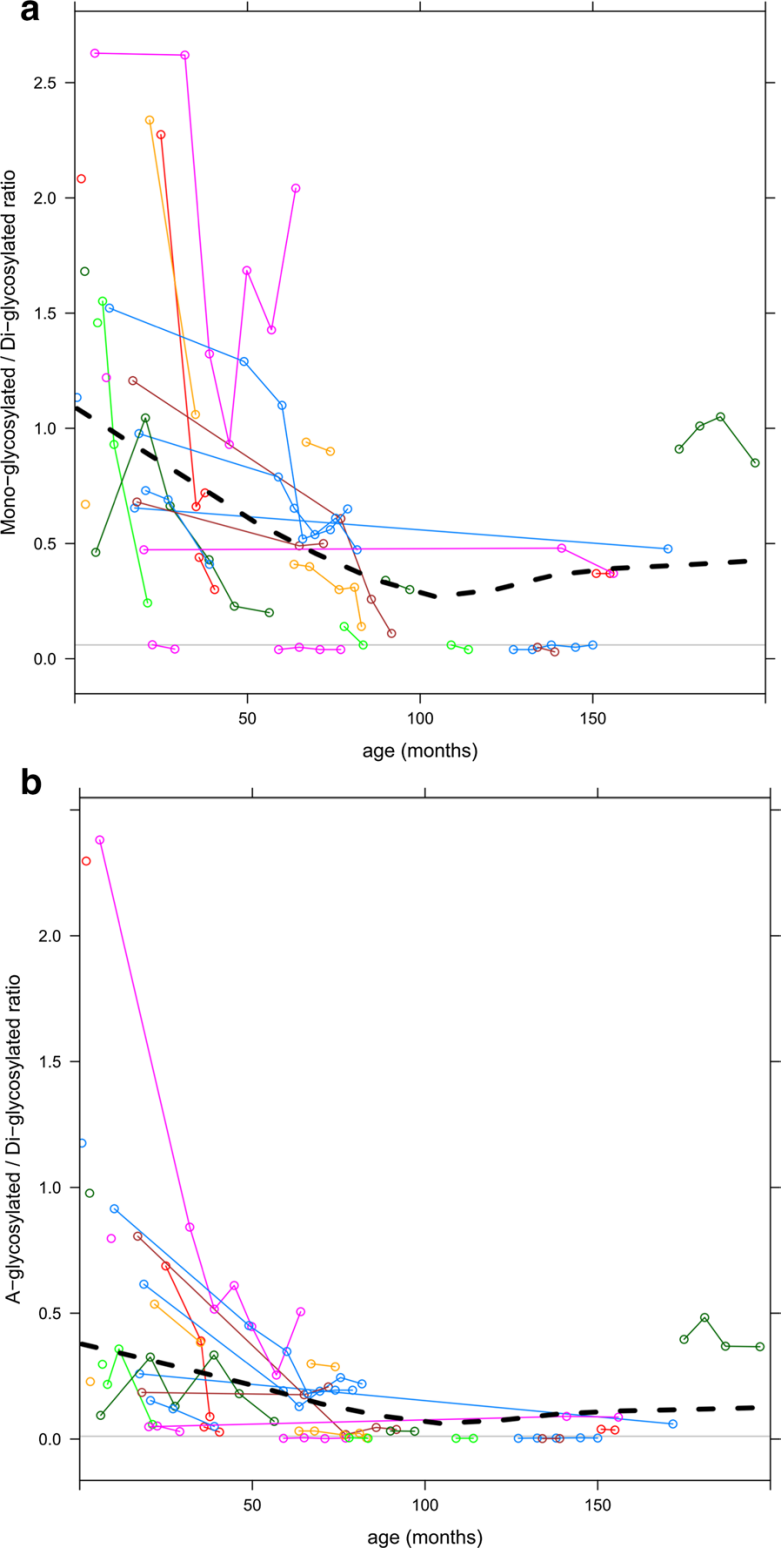
Measurements of transferrin glycosylation by MS. Each circle represents one measurement. Measurements in the same patient over time are connected by thin lines. A Loess regression curve is plotted a black dotted line. The horizontal grey line represents the upper limit of normal. Measurements include **a** ratio of mono-glycosylated (hypoglycosylated) transferrin to di-glycosylated (normal) transferrin, **b** ratio of a-glycosylated (non-glycosylated) transferrin to di-glycosylated (normal) transferrin

Results of our analysis are presented in Fig. 1. Over time, there is a clear improvement of transferrin glycosylation with a decrease of the mono-/di-glycosylated transferrin ratio from 0.76 ± 0.71 to 0.47 ± 0.45 (*P* < 0.001, Wilcoxon signed rank test, normal ratio value < 0.06) and of the a-/di-glycosylated transferrin ratio from 0.34 ± 0.51 to 0.18 ± 0.29 (*P* = 0.031, normal ratio value < 0.0111). This happens, without any treatment, in patients with their first carbohydrate deficient transferrin analysis before the age of 100 months as evident from the Loess regression (black dotted line) in Fig. 1, the same age category in which Taday et al. report improvement of glycosylation. We also note that our untreated cohort has 7 PMM2-CDG patients with similar improvements in transferrin glycosylation as reported for ‘responders’ by Taday et al. and 4 PMM2-CDG patients with at least one completely normal carbohydrate deficient transferrin analysis. Additionally, we are concerned that, given the inherent variability of carbohydrate deficient transferrin in PMM2-CDG represented in our data set and the even more frequent measurements reported by Taday et al., using the best tetra sialo value for analysis might introduce representation bias.

Unfortunately, we have not yet accumulated sufficient longitudinal data in our patients < 100 months old showing glycosylation improvements to assess whether they have subsequent worsening of glycosylation in later years as observed by Taday et al. after stopping the mannose.

## (ib) Improvement of biochemical variables

The authors also mention (but “*not all data shown”*) improvements of anti-thrombin III, Protein C, protein S and factor IX as well as transaminases on mannose therapy. This is not surprising as age-related improvements of many of these variables (aPTT, Factor XI, Antithrombin III, Protein C, AST and ALT) in PMM2-CDG without mannose therapy has been previously published [4]. The authors do not mention whether they see deterioration of these variables after discontinuation of mannose. Further study is needed to understand how these hematologic parameters change in the natural history of the disorder and how they may contribute to long term clinical outcomes in PMM2-CDG.

## (ii) Clinical improvement

Taday et al. provide illustrative vignettes regarding nerve conduction velocity, attempting to suggest clinical improvement with mannose therapy. PMM2-CDG retrospective natural history studies suggest stabilization of clinical course as patients get older with development of later onset of specific disease symptoms, including neuropathy [4]. While Taday et al. imply deterioration of the nerve conduction velocity is due to stopping mannose therapy, as an acknowledged later onset feature of the disease, it is difficult to imply causality from this observation alone.

Their study, as implemented, is actually unable to demonstrate clinical improvement as it lacks the ability to compare clinical outcome parameters before and after treatment and lacks a control group, as all of their patients were supplemented with mannose. Their analysis raises a number of concerns. To start, defining responders as those that demonstrate an increase of tetra sialo-transferrin by 50% of pretreatment levels is arbitrary without demonstrated functional correlate, and is a metric that 3 of their ‘responders’ fail to meet based on the provided supplementary material (patients 7, 10, and 16). Second, it is misleading to compare ‘responders’ versus ‘non-responders’, which differ by a number of characteristics, including, as already noted, the ‘responders’ group is composed exclusively of younger PMM2-CDG patients, while the ‘non-responders’ has several older PMM2-CDG patients at initiation of mannose supplementation. Third, paradoxically, the rate at which mannose ‘responders’ discontinue mannose is much higher in than ‘non-responders’. All but one ‘non-responder’ continue mannose supplementation despite lack of a biochemical response (~ 88%), while only 25% of ‘responders’ continued mannose.

## Priorities for advancing a therapy for PMM2-CDG

Historically there was initial enthusiasm for mannose supplementation in PMM2-CDG given dramatic improvement in MPI-CDG with mannose supplementation [5] and biochemical correction with mannose in PMM2-CDG patient-derived fibroblasts [6]. Additionally, an early mouse model of PMM2-CDG demonstrated rescue of embryonic lethality with prenatal mannose supplementation to pregnant dams [7], a finding reproduced in a more recent knock-in mouse model of the European p.F119L mutation, but not the European catalytically inactive p.R141H mutation [8]. A number of publications of small, short-term studies of mannose supplementation in humans with PMM2-CDG revealed uniformly disappointing results without biochemical or clinical improvement with enteral or intravenous mannose supplementation [9–12].

In light of the limited natural history of PMM2-CDG, the report by Taday et al., unfortunately fails to provide convincing evidence for significant clinical improvement with long-term mannose supplementation in PMM2-CDG. These patients suffer from significant lifelong deficits and are in need of significant improvements in therapy. We are concerned about the futility of spending time and effort to pursue additional studies of mannose in PMM2-CDG, where there is very little evidence for efficacy. We note that there have been a number of recent advances in approaches to treat PMM2-CDG [13], including: a recently reported open-label study demonstrating improvement in ataxia with acetazolamide [14], pharmacologic chaperones [15] or aldose reductase inhibitors identified through a drug-repurposing screen [16], and mannose-1-phosphate replacement therapies intended to bypass the PMM2-dependant biosynthetic step [13].

So where do we go from here? Given the sudden availability of multiple potential treatment approaches other than mannose for PMM2-CDG and the impossibility of performing well-powered, placebo-controlled, double blind studies for all of them, it is critical for professionals and families to prioritize (1) the treatment approaches with the strongest evidence of potential efficacy that are (2) most likely to result in meaningful improvements for patients. It is critical to work as a global community to subject these treatments to rigorous investigation so that unambiguous evidence of their efficacy can be obtained quickly. To maximally utilize these trials, it is critical that we amass a detailed, prospective natural history of PMM2-CDG, including changes in potential biomarkers, and clinical outcomes, such as through the Nijmegen pediatric CDG rating scale [17]. Use of transferrin as a biomarker of clinical efficacy is problematic given its trend toward normalization as patients age and its lack of correlation with clinical outcomes of importance to patients and families. Identification of new, robust biomarkers linked to clinical outcomes is essential in efforts to increase efficiency of clinical trials and potentially guide clinical management. Finally, a productive partnership with patient families will ensure that professional and patient goals are aligned to achieve meaningful therapeutic advances in PMM2-CDG. To this end, we invite all interested professionals to join in the hybrid virtual 5^th^ World CDG Conference May 14–16, 2021 to collaborate with patient advocacy groups to meaningfully move forward CDG therapeutics.

## Supplementary Information

**Additional file 1**. Source data: measurement of transferrin glycoyslation in individual patients.

## Data Availability

All data generated or analyzed during this study are included in this published article [and its supplementary information file].

## Abbreviations

CDG: Congenital disorders of glycosylation
PMM2-CDG: Phosphomannomutase 2 deficiency
MS: Mass spectrometry

## References

[1] Taday R, Grüneberg M, DuChesne I, Reunert J, Marquardt T (2020). Dietary mannose supplementation in phosphomannomutase 2 deficiency (PMM2-CDG). Orphanet J Rare Dis.

[2] Altassan R, Péanne R, Jaeken J, Barone R, Bidet M, Borgel D (2019). International clinical guidelines for the management of phosphomannomutase 2-congenital disorders of glycosylation: Diagnosis, treatment and follow up. J Inherit Metab Dis.

[3] Schiff M, Roda C, Monin M-L, Arion A, Barth M, Bednarek N (2017). Clinical, laboratory and molecular findings and long-term follow-up data in 96 French patients with PMM2-CDG (phosphomannomutase 2-congenital disorder of glycosylation) and review of the literature. J Med Genet.

[4] Witters P, Honzik T, Bauchart E, Altassan R, Pascreau T, Bruneel A (2019). Long-term follow-up in PMM2-CDG: are we ready to start treatment trials?. Genet Med.

[5] Niehues R, Hasilik M, Alton G, Körner C, Schiebe-Sukumar M, Koch HG (1998). Carbohydrate-deficient glycoprotein syndrome type Ib. Phosphomannose isomerase deficiency and mannose therapy. J Clin Invest..

[6] Panneerselvam K, Freeze HH (1996). Mannose corrects altered N-glycosylation in carbohydrate-deficient glycoprotein syndrome fibroblasts. J Clin Invest.

[7] Schneider A, Thiel C, Rindermann J, DeRossi C, Popovici D, Hoffmann GF (2011). Successful prenatal mannose treatment for congenital disorder of glycosylation-Ia in mice. Nat Med.

[8] Chan B, Clasquin M, Smolen GA, Histen G, Powe J, Chen Y (2016). A mouse model of a human congenital disorder of glycosylation caused by loss of PMM2. Hum Mol Genet.

[9] Grünert SC, Marquardt T, Lausch E, Fuchs H, Thiel C, Sutter M (2019). Unsuccessful intravenous D-mannose treatment in PMM2-CDG. Orphanet J Rare Dis.

[10] Kjaergaard S, Kristiansson B, Stibler H, Freeze HH, Schwartz M, Martinsson T (1998). Failure of short-term mannose therapy of patients with carbohydrate-deficient glycoprotein syndrome type 1A. Acta Paediatr.

[11] Mayatepek E, Kohlmüller D (1998). Mannose supplementation in carbohydrate-deficient glycoprotein syndrome type I and phosphomannomutase deficiency. Eur J Pediatr.

[12] Mayatepek E, Schröder M, Kohlmüller D, Bieger WP, Nützenadel W (1997). Continuous mannose infusion in carbohydrate-deficient glycoprotein syndrome type I. Acta Paediatr.

[13] Brasil S, Pascoal C, Francisco R, Marques-da-Silva D, Andreotti G, Videira PA (2018). CDG therapies: from bench to bedside. Int J Mol Sci..

[14] Martínez-Monseny AF, Bolasell M, Callejón-Póo L, Cuadras D, Freniche V, Itzep DC (2019). AZATAX: acetazolamide safety and efficacy in cerebellar syndrome in PMM2 congenital disorder of glycosylation (PMM2-CDG). Ann Neurol.

[15] Monticelli M, Liguori L, Allocca M, Andreotti G, Cubellis MV (2019). β-Glucose-1,6-bisphosphate stabilizes pathological phophomannomutase2 mutants in vitro and represents a lead compound to develop pharmacological chaperones for the most common disorder of glycosylation, PMM2-CDG. Int J Mol Sci..

[16] Iyer S, Sam FS, DiPrimio N, Preston G, Verheijen J, Murthy K (2019). Repurposing the aldose reductase inhibitor and diabetic neuropathy drug epalrestat for the congenital disorder of glycosylation PMM2-CDG. Dis Model Mech..

[17] Achouitar S, Mohamed M, Gardeitchik T, Wortmann SB, Sykut-Cegielska J, Ensenauer R (2011). Nijmegen paediatric CDG rating scale: a novel tool to assess disease progression. J Inherit Metab Dis.

